# The Risk of Nonsteroidal Anti-Inflammatory Drugs in Pediatric Medicine: Listen Carefully to Children with Pain

**DOI:** 10.3390/children8111048

**Published:** 2021-11-13

**Authors:** Olivier Mboma, Stefan Wirth, Malik Aydin

**Affiliations:** 1 Center for Child and Adolescent Medicine, Center for Clinical and Translational Research (CCTR), Helios University Hospital Wuppertal, Witten/Herdecke University, 42283 Wuppertal, Germany; olivier.mboma@helios-gesundheit.de (O.M.); stefan.wirth@helios-gesundheit.de (S.W.); 2 Laboratory of Experimental Pediatric Pneumology and Allergology, Center for Biomedical Education and Research, School of Life Sciences (ZBAF), Faculty of Health, Witten/Herdecke University, 58455 Witten, Germany

**Keywords:** ibuprofen, nonsteroidal anti-inflammatory drugs, acute kidney injury, pediatric

## Abstract

Over the last decades, the use of over-the-counter analgesics in the general population has increased in Germany. Ibuprofen is one of the most commonly used nonsteroidal anti-inflammatory drug (NSAID) and is frequently prescribed to children as an analgesic and/or antipyretic. Besides having a well-established safety and efficacy profile when used in appropriate doses, cases of NSAID-induced acute kidney injury (AKI) have been described in the pediatric population, particularly in the context of dehydration and in combination with other drugs. The ingestion of more than 400 mg/kg is associated with severe or life-threatening toxicity. This report is about two previously healthy adolescents, who developed AKI after taking high daily dose of ibuprofen as a pain reliever without any appropriate medical supervision. With these case reports, in addition to the side effect profiles of this analgesic, we would also like to present a certain therapeutic recommendation that we applied in these patients, and furthermore appeal to pediatricians to strictly set the indications for ibuprofen intake.

## 1. Introduction

Ibuprofen is one of the most commonly used over-the-counter nonsteroidal anti-inflammatory drug (NSAID), which is frequently prescribed to children as an analgesic and/or antipyretic [1,2,3]. Although this drug has a well-established safety and efficacy profile when used in appropriate doses, several authors warn against its use, as it can lead to acute kidney injury (AKI), previously called acute renal failure, especially in the context of dehydration and in combination with other drugs, including acetaminophen (paracetamol) [2,4,5,6]. The main mechanism of AKI caused by NSAIDs occurs through decreased kidney perfusion [7]. Mechanistically, nonselective NSAIDs inhibit the two isoforms of the enzyme cyclooxygenase (COX-1 and COX-2), which interferes with the conversion of arachidonic acid into prostaglandins E2, prostacyclins and thromboxanes, preventing the compensatory vasodilation response of prostaglandins [7]. While COX-1 mainly controls renal hemodynamics and glomerular filtration rate (GFR), COX-2 mainly affects salt and water excretion [8]. This leads to situations of volume deficiency, acute renal failure associated with the administration of NSAIDs, due to a decrease in renal plasma flow and GFR caused by the lack of prostaglandins [7,8]. Furthermore, NSAIDs can also cause AKI by inducing acute interstitial nephritis (AIN), which is characterized by the presence of inflammatory infiltrates and edema in the interstitium of the kidney [7,9]. In pediatrics, the daily dose of ibuprofen is prescribed based on the age and weight of the child with an optimal analgesic dose of 10 mg/kg body weight every 8 h with the maximum single dose and daily dose being 800 mg and 2400 mg [10]. Symptoms of severe ibuprofen toxicity are uncommon in children at doses less than 100 mg/kg ingested by history over the total course of treatment [11,12,13]. Ingestion greater than 400 mg/kg body weight is associated with a higher risk of severe or life-threatening toxicities including, but not limited to, gastrointestinal bleeding, thrombocytopenia, pulmonary edema, or severe acute kidney failure and metabolic acidosis [11,12]. Further treatment should usually be provided by primary supportive measures, as unfortunately no antidote is available [11]. Gastrointestinal decontamination with activated charcoal may be performed if a clinically significant ibuprofen overdose greater than 400 mg/kg has occurred within two hours [11].

Ingestion of less than 100 mg/kg is unlikely to require treatment and is safe to monitor at home as the elimination half-life does not appear to increase with overdose and symptoms do not appear later than four hours after ingestion [11,13]. For moderate intake (100–400 mg/kg of ibuprofen) or mild to moderate symptoms, the treatment should be based on clinical judgment [14].

Here, we report about two interesting cases of ibuprofen-induced AKI that were managed at the pediatric department of the Helios University Hospital Wuppertal, Witten/Herdecke University, Germany between April, and June 2021.

## 2. Clinical Presentation and Course of Case I°

A 17-year-old male presented with a two-day history of abdominal pain and vomiting. No diarrhea or fever were described. He reported a motorcycle accident that had occurred four days earlier and had sustained a Tossy-III fracture of the right shoulder, which was treated with a clavicle brace. A trauma spiral performed at the same day showed no further abnormalities.

Except for having overweight (weight 98 kg, height 186 cm, BMI 28 kg/m^2^), the physical exam was normal. Laboratory explorations showed elevated creatinine parameters at 2.4 mg/dL (normal range: 0.6–1.3 mg/dL) and an acute renal failure with a glomerular filtration rate (GFR) of 39 mL/min/1.73 m² (Table 1).

Using the ‘Kidney Disease: Improving Global Outcomes’ (KDIGO) criteria, the patient was at stage 2 with concordant results using the ‘Pediatric Risk, Injury, Failure, Loss, End Stage Renal Disease’ (pRIFLE) (Table 2).

**Figure 1:**
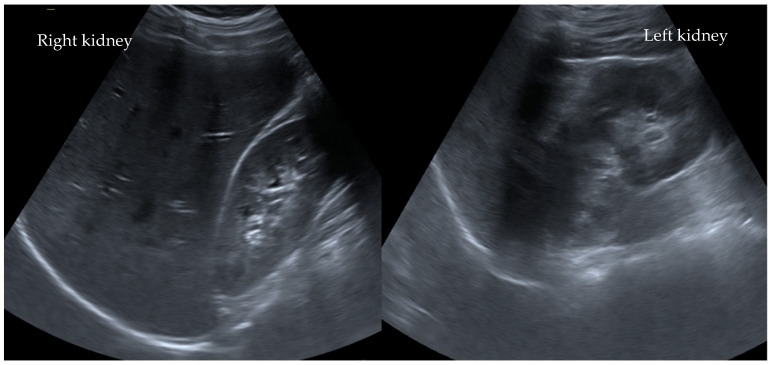
No parenchymal pathologies observed ultrasonographically in the kidneys after ibuprofen misuse in Case I°. Ultrasonographically, there are no parenchymal changes, medullary pyramids, especially sonomorphologically laterally symmetrical and inconspicuous with good corticomedullary parenchymal differentiation. The kidneys are normal in size. The resistance index is laterally symmetrical and normal for age.

**Figure 2:**
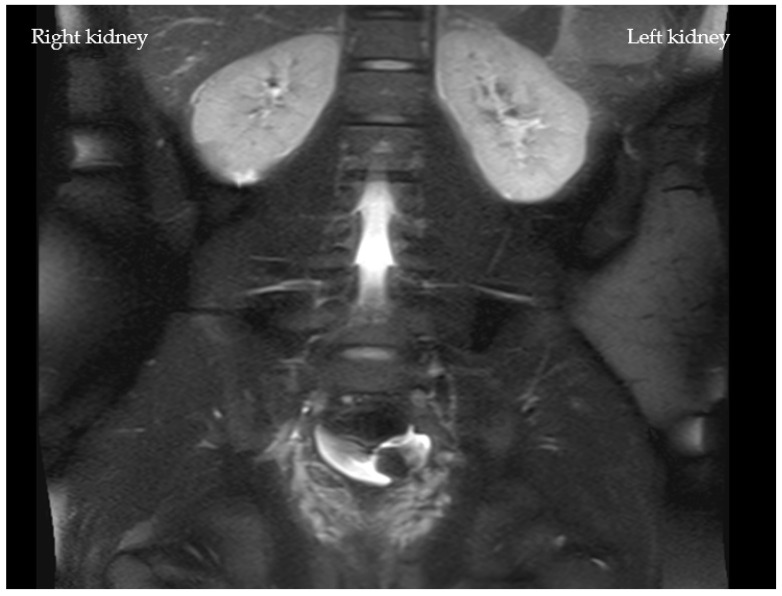
MRI of the abdomen of Case I° with a frontal cut of the two kidneys. An MRI of the abdomen showed a mildly enlarged appendix without surrounding edema and little free fluid in the pelvis. The kidneys showed mildly irregular signaling in the upper renal pole on both sides and the lower pole on the right side (cortical). Duplex sonography of the renal artery showed a laterally symmetrical resistance index normal for age.

In addition, both potassium and C-reactive protein (CRP) levels were highly elevated at 6.3 mmol/L and 4.0 mg/dL, respectively. All other laboratory tests were within the normal range. Interestingly, an abdominal ultrasound revealed no abnormalities (Figure 1). Due to the suspicion of an acute abdomen, elevated inflammatory parameters, and an increase in serum creatinine to 3.1 mg/dL (stage failure according to pRIFLE), an ultrasonography of the abdomen (Figure 1) was performed. An MRI scan revealed slightly irregular signaling in the upper renal pole region on both sides in the context of possible interstitial nephritis (Figure 2).

**Figure 3:**
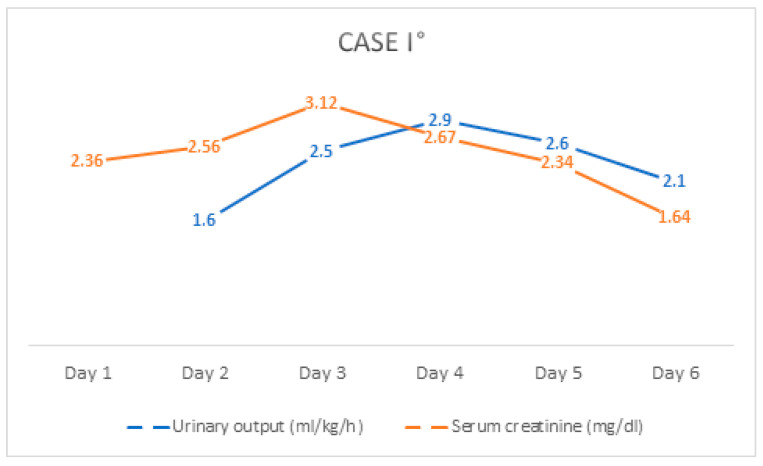
The course of serum creatinine value and urinary output of Case I° during hospitalization.

After a careful history, the patient noted that he had taken a substantial dose of ibuprofen (4 g; 41 mg/kg/d) for pain over four days. Subsequently, based on the history and laboratory findings, a diagnosis of ibuprofen-induced acute renal failure was made and therapy with intravenous fluid substitution was initiated. He also received antibiotic therapy with piperacillin/tazobactam for unclear acute abdomen and inflammatory parameters. He received dimenhydrinate for vomiting and additional pantoprazole for gastric protection. Since the inflammatory parameters regressed and no pathogens were detected in blood and urine culture, we discontinued antibiotic therapy after five days. The patient’s renal values and clinical condition also improved, so that he could be discharged on day seven. At follow-up, the patient was symptom-free and laboratory values normalized, so we consider him to have recovered to a *restitutio ad integrum* status (Figure 3).

## 3. Clinical Presentation and Course of Case II°

A 16-year-old female patient was transferred from an extern children’s hospital with severe bilateral flank pain for one day and vomiting. In the medical history, the girl stated that she had severe menstrual cramps. No other complaints were stated by the patient.

On physical examination, she had an obese nutritional status (weight 112 kg, height 169 cm, BMI 39 kg/m^2^) and complained of bilateral renal angle tenderness. The laboratory parameters revealed elevated creatinine parameters at 3.3 mg/dL (range: 0.5–1.1 mg/dL) and an acute renal failure with a GFR of 20 mL/min/1.73 m at the stage of failure using the pRIFLE classification and at the stage 3 with the KDIGO. In addition, she presented elevated potassium and CRP with 7.4 mmol/L and 4.5 mg/dL, respectively. All other laboratory tests were within the normal range (Table 3).

**Figure 4:**
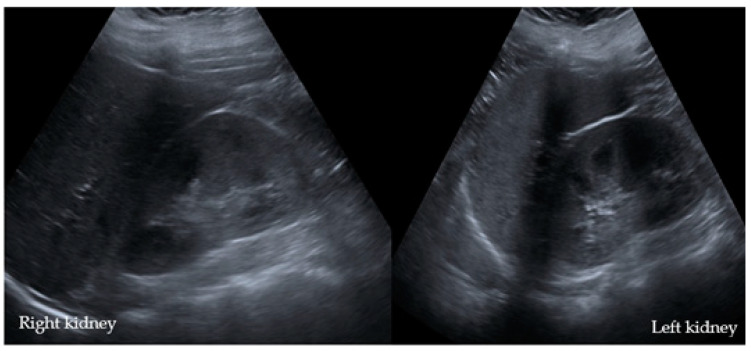
An ultrasonography of the kidneys of Case II°. Both kidneys presented mild echogenic accentuation of the parenchyma with consecutive increased corticomedullary differentiation.

Abdominal sonography showed a bilateral echo-rich region of the renal cortex on both sides (Figure 4).

**Figure 5:**
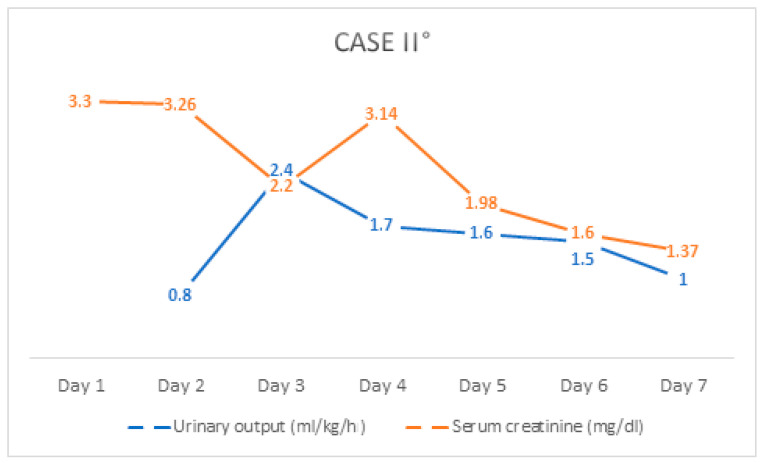
The course of serum creatinine value and urinary output of Case II° during hospitalization.

After a careful and detailed history, the patient reported that she had taken 12–14 tablets of ibuprofen (=46 mg/kg/d) a few days ago because of severe menstrual cramps. Considering the biochemistry, radiological findings, and history, a diagnosis of ibuprofen-induced AKI was made, and increased fluid substitution was performed. In the case of increased infection parameters and a suspicion of acute pyelonephritis, antibiotic therapy with cefuroxime and ampicillin was also administered and discontinued after inflammation had subsided. Urine culture grew enterococci with a bacterial count of 10^2^/mL, which was most indicative of contamination. To relieve pain, she received metamizole and acetaminophen, dimenhydrinate against vomiting and additional pantoprazole for stomach protection. Under the above-mentioned therapy, the general condition improved rapidly; the symptoms regressed, as did the retention parameters with decreasing serum creatinine level (Figure 5). She was discharged from the hospital on the seventh day with a pediatric follow-up appointment.

## 4. Discussion

We describe two adolescent inpatients who developed AKI despite not having renal disease or being treated concomitantly with other medications. Both patients were taking ibuprofen beyond the recommended daily dose for pain relief and without medical supervision. Within a few days, they experienced abdominal pain and vomiting as their main symptoms. The two patients showed no signs of hematuria, oliguria, or other symptoms that might have indicated renal impairment.

Biochemical analysis revealed at least a doubling of serum creatinine and elevated potassium levels in both adolescents, suggesting AKI. Based on this new information, a new medical history was obtained and asked whether potentially nephrotoxic substances, including ibuprofen, had been taken in the previous days. According to the pRIFLE criteria both did not pass the stage failure and the KDIGO stage 3. An MRI was performed with deteriorating GFR and unremarkable sonographic examination. A slightly irregular signal enhancement was seen, but interpretation was limited without contrast administration. Both patients received antibiotics due to elevated CRP parameters. In Case I°, we had a situation of acute abdomen without a clear cause with elevated inflammatory markers. After consultation with surgical colleagues, no diagnostic laparoscopy was performed and antibiotic therapy with piperacillin/tazobactam was administered instead, as bacterial infection was suspected. The girl in Case II° presented with a suspicion of pyelonephritis and therefore received ampicillin and cefuroxime, but in both cases, the antibiotic treatment was stopped after receiving the sterile culture results. Since both patients showed rapid clinical improvement, we decided not to perform further investigations, and no 24 h-urine, tubule function enzymes were analyzed nor was a renal biopsy performed to rule out AIN. This should be taken into consideration in case of persistent symptoms.

As a result of the timely diagnosis of NSAID-induced AKI, the disease healed within a week with recovered renal function, since the triggering agent was immediately discontinued, and intravenous fluid was administered. No unrelated kidney disease relapsed in any of the patients. At the last follow-up examination, none of the patients presented any further symptoms and the laboratory values had normalized (=*restitutio ad integrum*). Further nephrological follow-up was organized by the resident pediatrician.

Due to its often insidious, non-oliguric, or asymptomatic development, ibuprofen-induced AKI can be easily overlooked, particularly when serum creatinine levels are not determined [16]. Immediate discontinuation of the precipitating agent usually results in regression of the condition within 72 to 96 h, but may take up to a week [17]. It is important to recognize drug-induced AKI, as prolonged injury can lead to permanent scars of the kidney parenchyma unless the drug is stopped [18,19]. The most current and preferred definition for pediatric AKI is the KDIGO classification [15]. The pRIFLE criteria can also be used to determine the stage of the AKI and can help to predict morbidity and mortality [20]. Each definition has its own advantages and disadvantages, but it appears that the pRIFLE classification outperforms the other methods in predicting AKI in several pediatric patient populations, because it can diagnose a larger number of cases of mild AKI [15,20]. However, these classifications are highly correlated with outcomes such as mortality or length of ICU stay [15,21].

It is important to note that the gold standard for the diagnosis of AKI due to AIN is a renal biopsy [22]. The histopathological picture shows plasma cell and lymphocytic infiltration in the peritubular areas of the interstitium, usually with interstitial edema [22]. If a patient’s clinical condition does not improve even after discontinuation of NSAIDs and appropriate fluid therapy, AIN should be strongly suspected. In collaboration with pediatric nephrologists, a renal biopsy should be recommended unless contraindications exist. [22]. In addition, the creatinine-clearance in 24 h-urine, and the determination of tubular enzymes should be also performed.

The daily dose of ibuprofen is chosen based on the age and weight of the child. In this regard, the optimal analgesic dose of ibuprofen for oral administration is 10 mg/kg every 8 h and the cumulative daily dose should not exceed 30 mg/kg with a maximum single dose of 800 mg and daily dose of 2400 mg [10]. The risk for developing AKI appears to be dependent on high daily dose [4,16].

A retrospective chart review of children diagnosed with AKI conducted during an 11.5-year period revealed, that the prevalence of NSAID-associated AKI was up to 2.7%, of whom 67% presented with volume depletion [23]. In another prospective case-control study, the prevalence of AKI was 44% of children hospitalized with dehydration due to acute gastroenteritis [5]. They also noticed that severe volume deficiency is not needed to be present in children to develop ibuprofen-induced AKI. This is an important clinical finding, because milder degrees of dehydration are not always clearly evident [5], which have been the case in our two examples. Nevertheless, renal failure is an uncommon adverse effect of NSAID in the pediatric population when taken as recommended [24].

In most cases, AKI has a reversible course and responds benign to supportive measures and intravenous fluids, and renal replacement therapy is required in only a few cases [12]. When NSAID toxicity is suspected, the diagnosis is made based on the information on the exact medication, amount, and timing [14]. While determination of serum ibuprofen levels is of no clinical benefit in an acute situation, a plasma acetaminophen level should be determined in case of doubt, as there are a number of over-the-counter combination products containing ibuprofen and acetaminophen [12,14].

Symptoms of severe ibuprofen toxicity are uncommon in children at doses less than 100 mg/kg over the total course of treatment [11,12,13]. Ingestion greater than 400 mg/kg body weight is associated with a higher risk of severe or life-threatening toxicities [11,12].

It should be noted, however, that cases of NSAID-induced AKI with a dose of less than 100 mg/kg and simultaneous dehydration have been described [10,25].

Children who have taken less than 100 mg/kg of ibuprofen and remain asymptomatic with normal vital signs may be discharged after a four- to six-hour observation period and no further routine testing is needed [11,12,14]. In cases of acute intoxication, gastrointestinal decontamination with activated charcoal may be performed if a clinically significant ibuprofen overdose greater than 400 mg/kg has occurred within two hours [11]. In addition, laboratory tests, i.e., blood glucose, electrolytes, blood urea, creatinine, and complete cell count should be performed in these patients [11,12,14]. Patients with severe vomiting or signs of clinical dehydration should receive intravenous fluid rehydration to ensure good renal perfusion and urinary excretion [12]. In cases of life-threatening courses, intensive medical care may be needed [11,12,14].

## 5. Conclusions

Although renal complications appear to be rare in the pediatric population when NSAIDs are taken correctly, pediatricians should be made aware of this potential complication. With over the counter NSAIDs such as ibuprofen growing in popularity in recent years, proper education about the dangers of NSAIDs is particularly important, and doctors should always check that this is not the cause of an acute illness.

## Figures and Tables

**Table 1:** Laboratory findings from Case I°.

	Age- and Sex-Adjusted Reference Range *	Admission	Discharge
Leukocytes (/nL)	4.3–10.0	13.3	7.6
Hemoglobin (g/dL)	14.0–18.9	16.0	16.9
Erythrocytes (/pL)	4.5–5.9	4.8	5.1
Hematocrit (%)	41.0–53.0	43.8	45.6
MCV (fL)	80–96	90.7	90.3
MCH (pg)	28–33	33.1	33.5
Thrombocytes (/nL)	150–350	217	256
GPT (U/I)	10–50	23	-
GGT (U/I)	<66	32	-
Lactate (mmol/L)		1.5	-
CK-MB (U/I)	<24	13	-
Potassium (mmol/L)	3.5–5.1	6.3	4.2
Sodium (mmol/L)	135–145	136	139
Chlorid (mmol/L)	96–106	104	99
Calcium (mmol/L)	-	2.3	2.62
Creatinine (mg/dL)	0.6–1.3	2.4	1.2
GFR (mL/min/1.73 m^2^)	-	39	87
Urea (mg/dL)	<50	44	43
CRP (mg/dL)	<0.5	4.0	0.6

* Reference values are influenced by many variables, including patient population, age, gender, and laboratory methods used. MCV = mean corpuscular volume, MCH = mean corpuscular hemoglobin, GPT = glutamate-pyruvate transaminase, GGT = gamma-glutamyltransferase, CK-MB = creatine kinase myocardial band, GFR= glomerular filtration rate, CRP = C-reactive protein.

**Table 2:** Pediatric acute kidney injury definitions, adapted from Sethi et al. (2021) [15].

	Staging	Creatinine Criteria	Urine Output Criteria
**pRIFLE**	Risk (R)	eGFR decrease by ≥25% *	<0.5 mL/kg/h for 8 h
	Injury (I)	eGFR decrease by ≥50% *	<0.5 mL/kg/h for 16 h
	Failure (F)	eGFR decrease by ≥75% or <35 mL/min/1.73 m^2^ *	<0.3 mL/kg/h for 24 h or anuric for 12 h
	Loss (L)	Loss of renal function > 4 weeks	
	End-Stage (E)	End Stage Renal Disease (persistent failure > 3 months)	
**KDIGO**	1	SCr rise ≥ 0.3 mg/dL within 48 h or an increase in creatinine of ≥50% within 7 day	>0.5 and ≤ 1 mL/kg/h
	2	Increase in creatinine of ≥100%	>0.3 and ≤0.5 mL/kg/h
	3	Increase in creatinine of ≥200% or SCr ≥ 4 mg/dL or receipt of dialysis or eGFR < 35 mL/min/1.73 m^2^	<0.3 mL/kg/h

* eGFR (estimated glomerular filtration rate) calculated with Schwartz equation: Length (cm) × K (constant)/serum creatinine, SCr (serum creatinine).

**Table 3:** Laboratory findings from Case II°.

	Age- and Sex-Adjusted Reference Range *	Admission	Discharge
Leukocytes (/nL)	4.3–10.0	11.6	8.4
Hemoglobin (g/dL)	12.0–16.0	12.7	13.6
Erythrocytes (/pL)	4.0–5.2	4.3	4.7
Hematocrit (%)	36.0–46.0	36.5	40.1
MCV (fL)	80–96	84.3	86.2
MCH (pg)	28–33	29.3	29.2
Thrombocytes (/nL)	150–350	297	327
Potassium (mmol/L)	3.5–5.1	7.4	4.3
Sodium (mmol/L)	135–145	139	140
Chlorid (mmol/L)	96–106	110	105
Calcium (mmol/L)	-	1.7	2.62
Creatinine (mg/dL)	0.5–1.1	3.3	1.3
GFR (ml/min/1.73 m^2^)	-	20	57
Urea (mg/dL)	<50	31	43
CRP (mg/dL)	<0.5	4.5	0.9

* Reference values are influenced by many variables, including patient population, age, gender, and laboratory methods used. MCV = mean corpuscular volume, MCH = mean corpuscular hemoglobin, GFR = glomerular filtration rate, CRP = C-reactive protein.

## Data Availability

There is no research data, which has to be shared separately. In any case of further questions, the corresponding/senior authors may be contacted separately through e-mail.
